# Prevalence and correlates of valvular heart diseases in the elderly population in Hubei, China

**DOI:** 10.1038/srep27253

**Published:** 2016-06-02

**Authors:** Chang Shu, Si Chen, Tingting Qin, Zhen Fu, Tucheng Sun, Mingxing Xie, Li Zhang, Nianguo Dong, Ping Yin

**Affiliations:** 1 Department of Epidemiology and Biostatistics, School of Public Health, Tongji Medical College, Huazhong University of Science and Technology, Wuhan, Hubei, China; 2 Department of Cardiovascular Surgery, Union Hospital, Tongji, Medical College, Huazhong University of Science and Technology, Wuhan, Hubei, China; 3 Department of Ultrasound, Union Hospital, Tongji, Medical College, Huazhong University of Science and Technology, Wuhan, Hubei, China

## Abstract

We sought to determine the prevalence and correlates of valvular heart diseases (VHD) in the elderly population. The participants’ personal information, medical history, behavioral habits and clinical status were assessed by questionnaire, while the left ventricular dimensions, function and the presence and severity of VHD were evaluated by transthoracic echocardiography. This study analyzed the data of 3948 participants who were older than 60 years. Significant VHD was present in 1.93% of participants; the standardized prevalence of VHD among the elderly population in Hubei was 2.05% (95% CI: 1.61–2.49). The most frequent VHD was aortic regurgitation, followed by tricuspid regurgitation, mitral regurgitation and multiple valve diseases. Univariate analysis results indicated that compared with participants without VHD, those with VHD were older (p < 0.001), with a higher body mass index (BMI) (p < 0.001), were more likely to smoke (p = 0.04), and had higher rates of coronary artery disease (CAD) (p < 0.001) and arrhythmia (p < 0.001). The results of multinomial regression analysis of complex sampling indicated that combined mitral and aortic valve diseases were related to older age, male sex and smoking; CAD was associated with single left-sided VHD.

Valvular heart disease (VHD) is a common condition in clinical practices that is strongly connected to heart dysfunction and death. Although VHD occurs less frequently than coronary heart disease, heart failure, or hypertension, it remains an important cause of increased morbidity and mortality^1^.

In the past decades, the epidemiology of VHD has changed dramatically in parallel with socio-economic development and an increasing aging population^2^,^3^. Population-based studies show that valvular disease is frequent in industrialized countries where the decrease in the prevalence of rheumatic heart disease has been compensated for by the increase in the prevalence of degenerative valve disease^4^. This explains the important changes in the presentation of valvular disease, which now mainly affects predominantly older people. However, rheumatic heart disease remains the main etiology in developing countries^5^. The overall VHD prevalence in the USA is 2.5% with a wide age-related variation from 0.7–13.3%^6^. The prevalence increased significantly with age, from less than 2% before 65 years, to 8.5% between 65 years and 75 years, and 13.2% after 75 years. Similar age tendencies were also demonstrated in the Euro Heart Survey^7^.

Population research projects an imminent increase in the elderly population in China, expecting an estimated 234 million people aged 60 years or older by 2020; the number of people older than the age of 60 is expected to double by 2040^8^. This report indicates that China is an aging society. As a result of the aging Chinese population, the role of degenerative, age-related, VHD as a public health issue should be reconsidered. Particularly, data on the prevalence of VHD in older individuals are scarce and based mostly on in-hospital series, introducing an important selection bias. Therefore, this study assessed the prevalence of VHD in the elderly population using a population-based cross-sectional epidemiological survey.

The aim of the present study was twofold. First, the prevalence of VHD was determined in the elderly population. Second, the correlates of VHD were assessed in the study population.

## Method

### Ethical considerations

The study protocol was approved by the ethical committee of the School of Public Health, Tongji Medical College, Huazhong University of Science and Technology, Wuhan, China [No. (2012)13]. The methods used were in accordance with the approved guidelines and informed consent was provided by each subject.

### Study population

The Valvular Heart Disease Epidemiological Survey was conducted from September 2012 to December 2015 in four geographically distinct sites in Hubei Province, China: Wuhan, Jingzhou, Enshi and Shiyan. We adopted stratified cluster sampling to enroll the participants. To ensure a representative nature of the study sample, the population proportion among 4 cities was considered. In each city, we selected 3–5 sub-districts in urban areas and 2–3 towns in rural areas. We then enrolled 3–5 residential units in each sub-districts and 3–5 villages in each town. Eligible persons included all community-dwelling persons who were able to participate in all examination procedures were enrolled. All participants answered questions regarding quality of life, physical activity, other social factors, nutrition and medical and personal history. Physical examination and transthoracic echocardiography was performed after participants completed the questionnaire. The Valvular Heart Disease Epidemiological Survey included 20,006 participants who had been recruited in 2012–2015. Our study population included 3948 individuals from the Valvular Heart Disease Epidemiological Survey who were older than 60 years of age.

### Clinical evaluation

Clinical status was evaluated in all individuals using the questionnaire. The medical history was recorded, including history of coronary artery diseases, hypertension, diabetes mellitus, surgery etc. Blood pressure was measured using a sphygmomanometer, in a seated position, after at least five minutes of rest without having performed vigorous exercise during the preceding 30 minutes. We used the mean of the assessed systolic values and diastolic values. Body mass index (BMI) was calculated by measuring the weight and height. Cardiac murmur was auscultated with a stethoscope.

Echocardiographic studies on subjects with a cardiac murmur and/or clinical symptoms were performed by physicians using a Vivid-I/Q portable machine (Vingmed-General Electric, Horton, Norway). Standard gray-scale and color Doppler images were acquired at a depth of 16 cm at the parasternal (standard long- and short-axis images) and apical views (2-, 4-chamber and apical long-axis images). Data were stored for further off-line analysis. Left ventricular (LV) diameters, interventricular septal (end-diastolic) thickness and posterior wall (end-diastolic) thickness were measured from M-mode images obtained from the parasternal long-axis view; LV ejection fraction was derived using the Teichholz formula. The valvular assessment included evaluation of the function of the mitral, aortic and tricuspid valves. Color-Doppler echocardiography was performed after optimizing gain and Nyquist limit, and standard continuous and pulsed-wave Doppler recordings were acquired. Stenotic and regurgitant valve diseases were evaluated using semiquantitative and quantitative methods as recommended by the American Society of Echocardiography^9^,^10^. The severity of valvular stenosis was based on the valve area and the mean pressure gradient across the restrictive orifice^11^. In addition, the severity of valvular regurgitation was determined on a qualitative scale and classified as mild (grade 1), moderate (grade 2) and severe (grades 3–4), according to the current ACC/AHA guidelines for the management of individuals with valvular heart disease.

In this study, significant valvular diseases were defined as any mitral or aortic stenosis severity, moderate or severe mitral regurgitation, moderate or severe aortic regurgitation and moderate or severe tricuspid stenosis or regurgitation.

### Statistical analysis

Analyses were performed with SAS statistical software (SAS Institute Inc., release 9.2, USA). The continuous variables were expressed as the mean ± standard deviation, while non-continuous variables were expressed as proportions. Student’s t-test was used for the comparison of quantitative data; the chi-square test was used for the comparison of qualitative data. Multinomial logistic regression analysis of complex sampling was used to examine correlates of left-sided valve disease. Two-tailed p < 0.05 was considered statistically significant.

## Results

### Characteristics of participants

A total of 3948 participants older than 60 years were included in this survey. Table 1 shows the basic characteristics of participants. The average age was 66.92 ± 5.35 years. Mean BMI was 22.61 ± 3.70 kg/m^2^; according to the WHO report^12^, 13.37% were underweight, 63.65% had normal body weight, 21.18% were overweight and 1.8% were obese. Mean systolic and diastolic BP were 133.84 ± 21.15 and 79.88 ± 12.01 mm Hg, respectively. The majority (78.95%) of participants reported they were current or former smokers, while 20.64% participants reported drinking behavior. A family history of cardiovascular disease was present in 13.75% of participants.

### Prevalence of valvular heart disease

Table 2 shows the prevalence of VHD on transthoracic echocardiography for the total study group. VHD was observed in 76 (1.93%) individuals. Because there was a significant gender imbalance, with a male/female ratio of 1:1.70, we adjusted the prevalence of VHD by sex distribution of the Hubei Province 2010 Population census^13^. The adjusted prevalence of VHD was 2.05% (95% CI: 1.61–2.49).Significant left-sided valvular disease involving only one valve (mitral or aortic) was noted in 32 (0.81%) individuals, whereas 5 (0.13%) individuals had both mitral and aortic valve involvement. The most frequent VHD was aortic regurgitation (AR) (44 patients, 1.11%) followed by tricuspid regurgitation (TR) (42 patients, 1.06%), multiple valve diseases (41 patients, 1.04%) and mitral regurgitation (MR) (41 patients, 1.04%). The tricuspid stenosis (TS) was infrequent, observed in only 1 (0.03%) participant.

### Characteristics of patients with VHDs

Characteristics of patients with VHDs are detailed in Table 3. The mean age of the 76 patients was 68.40 ± 5.85 years and 50.00% of patients were male. The mean BMI was 22.71 ± 3.62 kg/m^2^; by the WHO report^12^, 11.84% were underweight, 57.89% had normal body weight, 27.63% were overweight and 2.63% were obese. The most common symptom was weakness (52.63%), followed by palpitations (50.00%) and dyspnea (48.68%). The LV end-diastolic dimensions and end-systolic dimension were 50.95 ± 7.99 mm and 20.27 ± 9.20 mm, respectively. The mean LV ejection fraction of patients was 56.94 ± 11.08%. Left ventricular aneurysm was infrequent; 26.32% of patients had pulmonary hypertension.

### Potential factors associated with VHD

The potential factors associated with VHD are presented in Table 4. Participants with VHD tended to be elder and were more likely to be male, and have a higher BMI than those without VHD (from p < 0.05 to p < 0.001). Smoking (current or former), arrhythmia and coronary artery disease (CAD) were more frequently observed in participants with VHD than those without VHD (from p < 0.05 to p < 0.001).

### Multinomial logistic regression

Tricuspid valve diseases (TVD) is often secondary to left-sided valvular disease^14^; in the current study, single-TVD was infrequent (only 4 patients observed). For these reasons, only left-sided valve disease was considered in the multinomial logistic regression analysis of complex sampling on correlates of left-sided valve disease, which is shown in Table 5. The multinomial variable was recoded into four categories: ‘participant without VHD’, ‘participant with single mitral valve disease (MVD)’, ‘participant with single aortic valve diseases (AVD)’ and ‘participant with both MVD and AVD’. The ‘participant without VHD’ category is the reference category in the model. The independent variables were participants’ characteristics (age, gender and BMI), behavior factors (smoking, drinking), coexistent disease (hypertension, diabetes, dyslipidemia, CAD, arrhythmia) and family history. The results indicated that male sex, smoking and older age were strongly associated with combined mitral and aortic valve diseases. The presence of CAD was significantly associated with single left-sided VHD.

## Discussion

Over the past several decades, a number of studies have focused on the prevalence of VHD; however, this prevalence was variable among the studies. A population-based study performed in the United States reported that the prevalence of VHD was 13.2% after the age of 75 years^6^. Nkomo *et al.* demonstrated high absolute rates of VHD (11.7%) in the elderly population, with MR and aortic stenosis (AS) being the most frequent valve diseases (7.1% and 4.6%, respectively)^15^. The prevalence rates of VHD in the current study were much lower than those reported in previous studies. These discrepancies in prevalence rates can be explained by the fact that the definitions of VHD were different between the current study and earlier studies. The definition of VHD used by this study was symptomatic significant VHD. However, in most previous studies, the definition of VHD is symptomatic and asymptomatic VHD. In terms of the types of the VHD, the results of the present study were in stark contrast to previous studies. Lebowitz *et al.* found the prevalence of AR to be 16.4% in 70–79 year old group^16^. Osnabrugge *et al.* analyzed the data from 7 studies and reported that the pooled prevalence of AS in the general elderly population (>75 years old) was 12.4%^17^. Iung’s study which included 5001 patients referred to the hospital, showed that AS was the more frequent (43%) than AR (13%)^7^. However, in current study, only 1 case of AS was observed and the prevalence of AS was much lower than AR. This result was consistent with Pan’s conclusion that AR is more prevalent than AS in the elderly Chinese population^18^. Furthermore, a compensatory mechanism exists to normalize the left ventricle wall stress by increasing the left ventricle wall thickness in patients with AS. Therefore, patients with AS may be asymptomatic for many years^19^. This compensatory mechanism is not, however, indefinite; patients with severe AS are more likely to develop symptoms within 3 to 5 years^20^. When symptoms arise, surgical intervention is required because the average survival is only 2 to 3 years^21^. This poor clinical tolerance to AS may explain the higher prevalence of AS in inpatient-based studies. Choong *et al.* found MR in 34% of subjects over 60 years old^22^. The prevalence of mitral stenosis (MS) among hospital patients was 0.44% in the study of Ukita *et al.* conducted in Japan^23^. There are few data concerning the epidemiology of TVD. Singh’s study addressed the prevalence of TR reporting rates of 16% for mild TR and 0.8% for moderate TR^24^. Whereas most of aforementioned studies’ populations consisted of hospitalized patients, the current study sample was sampled from the general population. Because of the different clinical characteristics, the prevalence rates of various forms of VHD are lower than in previous studies. In addition, most previous studies were not contemporary to the current study, which also led to difficulties in comparing the results of this study to previous studies. Therefore, we may speculate that the prevalence of asymptomatic VHD is actually higher and that regular physical and ultrasound examinations are important for patients with VHD.

The factors found to be associated with combined mitral and aortic valve diseases were male sex, smoking, and older age, while the factor associated with single left-sided VHD was CAD.

Whether there are gender differences in VHD remains unclear. Podolec *et al.* reported that senile degenerated VHD was more often observed in old females^25^. An independent study of 815 patients by Boon *et al.* found AVD to be associated with female sex^26^. In contrast, the Cardiovascular Health Study reported that male sex was risk factor for AVD^27^. Other studies have suggested that VHD was not significantly related to gender^28^,^29^. In present study, only combined mitral and aortic valve diseases associated with male sex. Any relationship between gender and VHD is remains unclear and it requires further research.

Most epidemiological studies confirmed that smoking is significantly related to VHD, and our results support those previous studies. This could be explained by the fact that smoking may increase the risk for AVD through mechanisms analogous to those postulated for atherosclerosis, including adverse effects on endothelial permeability and lipoprotein oxidation. According to the literature, nicotine could induce mast cell activation. Furthermore, nicotine and acetaldehyde are both capable of directly inducing TGF-β1 mRNA expression in cultured human skin fibroblasts^30^, which could accelerate valvular stenosis. However, unlike other factors (such as age or, gender), smoking is a preventable risk factor. Jiang’s study reported that for light ex-smokers, early smoking cessation could delay or prevent the aortic calcification process and that the risk of aortic calcification decreased with the longer duration of cessation^31^. Therefore, actively quitting smoking is of great significance for patients with VHD in terms of both management and prevention.

In the study by Bozbas *et al.*, out of 346 patients who underwent surgery due to rheumatic heart disease, 218 (63%) underwent coronary angiography, and 18.8% of the patients were found to have CAD^32^. Chun *et al.* also reported that of 82 patients with MS who underwent CAG, 21 patients (26%) were found to have CAD^33^. Similar studies on patients with aortic stenosis reported a 56% prevalence rate for CAD^34^,^35^. In the present study, 28.57% of patients with single MVD, and 53.85% of the patients with single AVD had CAD, a finding similar to that reported in the literature. Emden’s study indicated that the atherosclerotic process played the role in the etiology of aortic stenosis and CAD played a major role in the etiology of MR^36^. Sclerosis or stenosis frequently occurred in multiple coronary artery branches with age and they could cause myocardial ischemia, reduce myocardial compliance and detract systolic and diastolic function. These abnormalities finally accelerate the damage to heart valves. The activity dysfunction due to cardiac valve calcification that was caused by CAD also accelerates coronary artery ischemia. This sets up a vicious circle and aggravates the disease state in the elderly. Therefore, strengthening the prevention of CAD in the clinical setting has positive significance for reducing the incidence of VHD.

As noted in previous studies, age was an independent risk factor of VHD; however, our results were not completely consistent. In our study, age was only associated with combined mitral and aortic valve diseases. Previous studies focused on the elderly mainly detected the risk factors of senile degenerative heart valve diseases or calcified valvular heart diseas. In the present study, the etiology of VHD was varied. Except for degenerative VHD and calcified VHD, the etiology of VHD in our study may have included rheumatic VHD, congenital VHD, and iatrogenic VHD. These factors may have masked the influence of age.

### Study limitations

In this study, we took multiple measures to ensure evaluation of all the patients with VHD. All of the investigators were well trained in cardiac auscultation; every participant with suspected murmur, VHD-related history, or VHD-related symptoms were suspected to have VHD and were evaluated by a cardiologist and with echocardiographic evaluation. Nevertheless, because echocardiographic evaluation was not performed in participants in the sample, the prevalence of valvular heart disease may have been underestimated.

## Additional Information

**How to cite this article**: Shu, C. *et al.* Prevalence and correlates of valvular heart diseases in the elderly population in Hubei, China. *Sci. Rep.* **6**, 27253; doi: 10.1038/srep27253 (2016).

## Figures and Tables

**Table 1:** Characteristics of participants.

Characteristics	Mean ± SD or N (%)
Age (y)	66.92 ± 5.35
Male gender	1463 (37.06)
BMI (kg/m^2^)	22.61 ± 3.70
SBP (mmHg)	133.84 ± 21.15
DBP (mmHg)	79.88 ± 12.01
Smoking (current or former)	3117 (78.95)
Drinking	815 (20.64)
Family history of cardiovascular disease	543 (13.75)

**Table 2:** Prevalence of Valvular heart disease.

	N	Prevalence (95%CI), %	Standardized Prevalence (95%CI), %
Valvular heart disease	76	1.93 (1.5–2.35)	2.05 (1.61–2.49)
Mitral valve disease	49	1.24 (0.90–1.59)	1.37 (1.01–1.73)
Mitral regurgitation	41	1.04 (.072–1.35)	1.17 (0.83–1.50)
Mitral stenosis	11	0.28 (0.11–0.44)	0.25 (0.10–0.41)
Aortic valve disease	48	1.22 (0.87–1.56)	1.34 (0.98–1.70)
Aortic regurgitation	44	1.11 (0.79–1.44)	1.24 (0.90–1.59)
Aortic stenosis	7	0.18 (0.05–0.31)	0.13 (0.02–0.24)
Tricuspid valve disease	42	1.06 (0.74–1.38)	1.14 (0.81–1.47)
Tricuspid regurgitation	42	1.06 (0.74–1.38)	1.14 (0.81–1.47)
Tricuspid stenosis	1	0.03 (0.00–0.07)	0.03 (0.00–0.07)
Multiple valve disease	41	1.04 (0.72–1.35)	1.17 (0.83–1.50)

3948 participants included.

**Table 3:** Characteristics of patients with valvular heart disease.

	mean ± SD or n (%)
Demographics	
Age (y)	68.40 ± 5.85
Male gender	38 (50.00)
BMI (kg/m^2^)	22.71 ± 3.62
Symptom	
Dyspnea	37 (48.68)
Palpitation	38 (50.00)
Syncope	17 (22.37)
Stenocardia	15 (19.74)
Throbbing sensation in neck	3 (3.95)
Edema in lower extremities	23 (30.26)
Echocardiography	
LV ejection fraction (%)	56.94 ± 11.08
LV end-diastolic dimension (mm)	50.95 ± 7.99
LV end-systolic dimension (mm)	20.27 ± 9.20
Left ventricular aneurysm	1 (1.32)
Pulmonary hypertension	20 (26.32)

76 patients included.

**Table 4:** Univariate analysis of potential factors associated with VHD.

	Participants without VHD (n = 3872) mean ± SD or n (%)	Participants with VHD (n = 76) mean ± SD or n (%)	*P* Value
Demographics			
Age (y)	67.41 ± 5.35	68.40 ± 5.85	<0.0001
Gender			
Male (n = 1463)	1425 (36.80)	38 (50.00)	0.0183
Female (n = 2485)	2447 (63.20)	38 (50.00)	
BMI	22.61 ± 3.70	22.71 ± 3.62	<0.0001
Behavior			
Smoking (current or former) (n = 3117)	3050 (78.77)	67 (88.16)	0.0468
Drinking (n = 815)	800 (20.82)	15 (19.74)	0.8183
Coexistent disease			
Hypertension (n = 1304)	1278 (33.06)	26 (34.21)	0.8324
Diabetes (n = 324)	319 (8.25)	5 (6.58)	0.5991
Dyslipidemia (n = 290)	282 (7.29)	8 (10.53)	0.2852
Coronary artery disease (n = 356)	336 (8.69)	20 (26.32)	<0.0001
Arrhythmia (n = 244)	228 (5.90)	16 (21.05)	<0.0001
Family history of cardiovascular disease (n = 543)	534 (13.94)	9 (11.84)	0.6014

**Table 5:** Logistic regressions.

	Wald χ^2^	*P* Value	OR	95% CI of OR
Single MVD				
CAD	4.7791	0.0288	4.560	1.170–17.776
Single AVD				
CAD	5.7267	0.0167	5.262	1.351–20.503
Combined mitral and aortic valve diseases				
age	6.9965	0.0082	1.077	1.019–1.137
Male	14.4323	0.0001	4.811	2.139–10.820
Smoking (current or former)	5.5689	0.0183	15.758	2.086–119.021
